# What can we learn from problem-based learning tutors at a graduate entry medical school? A mixed method approach

**DOI:** 10.1186/s12909-018-1214-2

**Published:** 2018-05-04

**Authors:** Diane O Doherty, Helena Mc Keague, Sarah Harney, Gerard Browne, Deirdre McGrath

**Affiliations:** 1 Graduate Entry Medical School, University of Limerick, Limerick, Ireland; 2 School of Medicine, University of Central Lancashire, Preston, United Kingdom

**Keywords:** Problem-based learning, Tutor experience, Small group learning, Graduate entry medicine

## Abstract

**Abstract:**

Problem-based learning (PBL) has been adopted by many medical schools as an innovative method to deliver an integrated medical curriculum since its inception at McMaster University (Dornan et al., Med Educ 39(2):163–170, 2005; Finucane et al., Med Educ 35(1):56–61, 2001; Barrows, Tutorials in problem-based learning: A new direction in teaching the health professions, 1984). The student experience in PBL has been explored in detail (Merriam, New Directions for Adult and Continuing Education 89: 3–13, 2001; Azer, Kaohsiung J Med Sci 25(5): 240–249, 2009; Boelens et al., BMC Med Ed 15(1): 84, 2015; Dolmans et al., Med Teach 24(2):173–180, 2002; Lee et al., Med Teach 35(2): e935-e942, 2013) but the tutors who facilitate PBL have valuable insight into how PBL functions and this aspect has not been extensively researched.

The integrated curriculum for years 1 and 2 at the Graduate Entry Medical School at the University of Limerick is delivered though problem-based learning (PBL). This programme requires collaborative teamwork between students and the tutors who facilitate small-group tutorial sessions. All PBL tutors at GEMS are medically qualified, with the majority (68%) currently working in clinical practice.

**Methods:**

A mixed-methods approach was adopted, utilising two surveys and follow-up focus groups to fully understand the tutor experience. Thirty-three tutors took part in two online surveys with a response rate of 89%. Thirteen tutors participated in two focus groups. Descriptive analysis was completed on survey data and thematic analysis on focus group discussions which highlighted five main themes.

**Results:**

Tutors reported challenges with managing group dynamics, development of confidence in tutoring with experience and a willingness to learn from peers to improve practice. Findings are in keeping with previously published work. Results also identified several less commonly discussed issues impacting student engagement in PBL including the use of mobile device technology, unauthorised access to learning objectives and PBL cases, and the importance and need for professional development amongst tutors, including the impact of tutoring on clinical practice. This study revealed that experienced tutors spend considerable time preparing for PBL tutorials in the basic sciences and that this input is rewarded by the benefits it brings to their clinical practice.

**Conclusions:**

Understanding PBL from the tutor’s perspective reveals valuable insights which can inform ongoing tutor development and support. Limited research exists in the area of PBL tutor’s experiences which may be of interest to medical educators, clinicians and the wider medical community. Findings highlight the value of shared tutor experiences as a resource that can be capitalised on to benefit both novice and experienced tutors.

**Electronic supplementary material:**

The online version of this article (10.1186/s12909-018-1214-2) contains supplementary material, which is available to authorized users.

## Background

This educational philosophy of problem-based learning (PBL) in healthcare integrates basic science and clinical skills teaching in the context of medical case studies and is particularly suited to the adult education setting of a graduate entry medical school [1–3]. This active ‘learner-centred’ approach to education that is integral to PBL has transformed the role of the tutor and faculty member into a deeper, facilitative position, providing ‘scaffolding’ to give structure and support to students whilst allowing self-directed learning [4]. The PBL tutor has a key role in influencing the successful outcome of PBL for students [5–7] and many studies have explored the skills and characteristics that distinguish ‘good’ PBL tutors [6, 8].

Much has been researched and written about the theory of PBL and the factors that influence its outcomes, such as tutor expertise [9–13], a belief in the rationale of PBL [14–16] and an ability to manage and maintain good group dynamics [8–13]. PBL programs vary widely in their practical implementation with differences in tutor background and qualifications, and curriculum design, making it difficult to compare PBL between different institutions and also to evaluate the educational outcomes of PBL compared to traditional curricula [17, 18]. It has also been recognised that, by definition, PBL occurs in small-group tutorials ‘behind closed doors’ [19] and that the practice may not always be consistent with the theory and ideals of the original educational model [20].

In this study, we set out to open the doors on PBL in the Graduate Entry Medical School at the University of Limerick where the hybrid PBL curriculum for Years 1 and 2 has been running for 10 years (See Additional file 1). The tutors who facilitate PBL have shared their exclusive vantage point on what works well in PBL and on their skills and strategies to deal with challenges to the success of PBL. At the University of Limerick, all of our tutors are medically qualified, with the majority in clinical practice, and this removes tutor background as source of variance [10], in addition to enhancing the tutors’ social and cognitive congruence with their students [8, 10]. Previous studies have identified that ‘cracks’ can appear in a long-running PBL curriculum without attention to the importance of maintaining the curriculum, promoting student engagement, adherence to process and ongoing tutor support and development [5]. Moust et al. [21] have also highlighted the potential for gradual divergence from PBL principles and process to impede student learning.

Here we addressed tutor experience as an under-researched and underrepresented source of information about the factors affecting PBL practice and outcomes [22]. The results of this study provide valuable insights into bridging the gap between PBL practice and theory and are generalisable to other PBL programs. Researching the tutors’ perspective has yielded unique information about student engagement with PBL, identifying how little reported issues such as the use of mobile devices, access to PBL cases and learning objectives, and milestones in the academic year negatively affect the PBL process. Our findings also challenge the idea that facilitating PBL is a passive process/or support the idea that facilitating PBL is a demanding/active process [5, 23], with evidence that tutors make a considerable time investment in preparing to facilitate PBL in the basic sciences and that, interestingly, this is rewarded by the positive benefits it brings to their clinical practice.

In addition to the impact of tutoring on their professional development as clinicians, the tutors in this study also displayed a willingness to develop their experience as educators; to learn from the shared experiences in their community of practice to ensure that their own practice was aligned with the theory and best practice in PBL. The information learned from experienced tutors can be used by PBL curriculum leaders to inform tutor support and development and to implement strategies to improve student engagement. In this way, we can enhance the experience of PBL for both students and tutors and help to ensure that students derive the optimal learning outcomes from PBL programs.

## Methods

This study adopted a mixed methods approach, utilising both quantitative and qualitative research methods – surveys and focus groups. In using a mixed methods approach it allowed researchers to harness the strengths of both qualitative and quantitative data, as argued by Tariq & Woodman [24] which is necessary when seeking to understand complex group dynamics. It also allows integration of results which offers readers confidence in the results and conclusions drawn [25]. The study was approved by the Education and Health Science Faculty Ethics Committee (2016_06_23_EHS).

### Quantitative

The study took place between October and December 2016. Data was collected via two online surveys with 33 tutors over the course of one semester.

### Qualitative

Two follow up focus groups (*n* = 13) took place based at the GEMS in December–January 2017.

#### Data collection

Non-probability sampling, namely purposive, was used to select participants. All PBL tutors (*n* = 33, excluding one of the authors) were sent an invitation to take part in this study. In order to gain a wider understanding of the different aspects of being a PBL tutor longitudinally, tutors were surveyed in two stages across two modules. Tutors may have had a negative experience in one module and a positive one for the other so it was deemed appropriate to gauge interactions and experiences twice in a semester. The online survey used in this study (see Additional file 2) was informed by subject matter experts and with input from experienced tutors based on a pilot survey. Feedback was examined in the following subject areas:

1. The process of problem-based learning for their group
2. Small group working and group dynamics
3. Tutor and group interaction
4. Curriculum content and ideas for improvement and innovation

Participants were aware of the voluntary nature of these surveys and confidentiality was assured. A link to the online survey was circulated to tutors at the end of each module by an independent gatekeeper. The survey was anonymous with completion implying consent. Data was compiled following the completion of both surveys. A number of contextual questions were asked which assessed each participant’s educational background, number of years facilitating PBL etc. The response rate overall was 89%. An overview of tutor background and teaching is shown in Table 1.

**Table 1:** Tutor demographics overview

	Percent
Tutor demographics	
12–18 months experience teaching as a PBL tutor	30%
> 2 years’ experience teaching as a PBL tutor	63%
% Year 1 tutors	44%
% Year 2 tutors	56%
Background: General Practice (GP, primary care)	71%
Background: Others (Pathology, Psychiatry, Anaesthesia & Oncology)	29%
Tutors still working clinically	68%
Clinical work: 5–10 sessions per week	35%
Tutor teaching experience	
• Last attended PBL training	25% in 2016, 22% in 2015, 22% in 2014
• Completed formal postgraduate education (MSc Med Ed, Dip Clinical Education)	20%
• Tutors who facilitated > 10 sessions per module	85%
• Facilitated module previously	93%
• < 60 mins preparation for sessions	10%
• 60–90 min preparation for sessions	20%
• 90–120 min	14%
• > 120 mins preparation for sessions	53%

(*n* = 59) Data are representative of two surveys completed

Focus groups allowed participants the opportunity to disclose any thoughts, feelings and negative experiences in a safe, non-judgemental environment. It also allowed authors to probe more deeply into the attitudes and experiences of tutors with respect to PBL. It was anticipated that the focus groups would also mitigate against the possibility of social desirability bias which can arise with surveys [26]. Focus groups were audio recorded for transcription. The group facilitator (DOD) was experienced in qualitative research methods and acted as an impartial member of staff as she was not engaged with teaching, tutors or other PBL staff. A focus group guide (see Additional file 3) was used by the facilitator which had been reviewed by the authors.

Volunteer sampling was used to gain participants for focus group sessions. Tutors who had completed surveys were invited to participate in a focus group and provided with information sheets via an email from an independent gatekeeper. The first focus group consisted of five tutors and the second eight tutors. All participants provided their written consent and were aware they could withdraw from the group if they wished and assured that all the data collected would be anonymised upon analysis and used strictly for the purpose of this study.

#### Data analysis

The statistical package SPSS version 22 for Windows was used for quantitative data analysis of surveys. Open-ended survey data was analysed qualitatively in NVivo 10 using thematic analysis to identify any issues to be further explored in focus groups.

Audio recordings of the focus groups were transcribed verbatim by a third party company to reduce bias in conjunction with NVivo 10, which was used to analyse the qualitative data using thematic analysis. Authors adopted Braun & Clarke’s [27] model for thematically analysing qualitative data. Thematic mapping allowed the coding and several reviews of themes by researchers. Researchers started coding the data using an inductive approach allowing for the identification of categories as they emerge from the data, revisiting data again and coding these into themes using phrases, experiences and thoughts [28]. All identifying information was removed with synonyms used to protect the identity of participants. The principal authors reviewed the final nodes and themes and a consensus was reached.

## Results

### Quantitative study

An overview of relevant quantitative results are referenced in Table 2 and items discussed further below.

**Table 2:** Overview of survey results

Tutor reflective practice			
Encouraged group to reflect on group dynamics		Every session	8%
		Weekly basis	29%
		Fortnightly	34%
		Once per module	24%
		*Missing Data*	5%
Self-reflection on own performance		After every session	39%
		On a weekly basis	42%
		Fortnightly	4%
		Once per module	2%
		*Missing Data*	3%
Group dynamics			
Input to maintain good group dynamics		‘Quite a bit’	27%
		‘Some’	48%
		‘A little bit’	17%
		‘Almost none’	3%
		*Missing Data*	5%
Group worked well together		‘Extremely satisfied’	39%
		‘Quite Satisfied’	47%
		‘Moderately Satisfied’	7%
		‘Slightly Satisfied’	2%
		*Missing Data*	5%
Students came to tutor with concerns		‘Never’	61%
		‘Very Often’	2%
		‘Often’	2%
		‘Sometimes’	5%
		‘Once in a while’	25%
		*Missing Data*	5%
Experienced difficulties in PBL sessions		Yes	20%
		No	75%
		*Missing Data*	5%
Relevance to clinical practice			
Module relevance for newly qualified doctors		‘Very relevant’	68%
		‘Quite relevant’	24%
		‘Moderately relevant’	5%
		*Missing Data*	3%
Cases reflective of up to date clinical practice		‘Very reflective’	29%
		‘Quite reflective’	61%
		‘Moderately reflective’	7%
		*Missing Data*	3%
PBL experience influenced tutor as a clinician		Yes	75%
		No	22%
		Missing Data	3%
Experience facilitating modules		‘Very positive’	37%
		‘Positive’	56%
		‘Neither positive or negative’	4%
		*Missing Data*	3%
Year-specific facilitation			
		Y1 Tutors	Y2 Tutors
Cases generating discussion	‘A great deal’	42%	42%
	‘Quite a bit’	42%	52%
	‘Some’	12%	3%
	*Missing Data*	4%	3%
Module relevance for newly qualified doctors	‘Very relevant’	42%	88%
	‘Quite relevant’	46%	6%
	‘Moderately relevant’	8%	3%
	*Missing Data*	4%	3%

(*n* = 59) Data are representative of two surveys completed

#### Time spent preparing for sessions

**Fig. 1:**
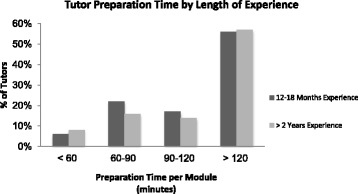
Tutor preparation time by length of experience. Data are representative of two surveys completed

Figure 1 illustrates the time that tutors devoted to preparing for PBL. Experienced tutors spent similar amounts of time on preparation with 56% of tutors with > 2 years’ experience and 55% of tutors with 12–18 months experiences spending > 120 min on preparation.

The reflective nature of PBL practice is also important as part of the PBL process for both staff and students. As outlined in Table 2, the majority of tutors discussed ground rules with students once per module, but only 29% encouraged the group to reflect on group practices on a weekly basis. Over a third of tutors also reflected on their own performance after every session.

#### Group dynamics

The issue of group dynamics has been extensively researched in the area of problem-based and small group learning. Table 2 summarises the tutors’ perceptions of the amount of input required to maintain good group dynamics with 75% reporting “quite a bit” or “some” interaction required and 86% reporting satisfaction with group performance. The focus group discussion did not completely reflect this high satisfaction rate but this can be explained by fact that data collected via surveys asked for opinions on specific modules just covered whereas focus group discussion referred to tutors’ experiences over a longer time period.

#### Group challenges faced in PBL sessions

Survey results showed that 20% of tutors experienced challenges in the PBL sessions during the study period and that the most common difficulties arose with timid and dominant group members, the use of prohibited electronic devices during sessions and pre-formed / dysfunctional groups.

#### Year-specific facilitation

**Fig. 2:**
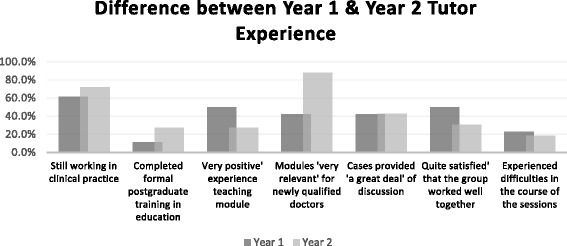
Comparisons of Y1 & Y2 tutors

Survey data (Fig. 2) revealed that Year 1 tutors reported more difficulties in sessions but also noted having a more positive experience compared to Year 2 tutors. Both tutor groups had equivalent lengths of service so this does not explain the observed difference. Year 2 tutors clearly deemed the Year 2 modules to be ‘very relevant’ for newly qualified doctors (88%), compared to only 42% tutors having the same opinion about the Year 1 modules. This reflects the gradual shift from fundamental basic science learning objectives in Year 1 to more clinically-focussed outcomes for Year 2.

#### Benefits to clinical practice

As outlined in Table 2, overall 68% of tutors (Year 1 and Year 2) felt that the modules facilitated during the survey period were ‘Very relevant’ for newly qualified doctors, with 61% also noting that the cases were ‘Quite reflective’ of up-to-date clinical practice. It is likely that the relevance of the PBL curriculum to clinical practice underlies the survey data showing that 75% of tutors felt their experience as a PBL tutor had influenced them as clinicians.

As outlined in Table 2, survey data showed that 38% of tutors felt they had a ‘Very positive’ experience facilitating the modules and 56% felt that the experience was ‘Positive’.

### Qualitative study

#### Thematic analysis

Data collected from focus groups was coded and analysing using a grounded theory approach and thematic analysis. Five key themes were identified with the input of three researchers, which aimed to reduce bias and aid in analysis.

##### 1. Preparation by tutor

Significant themes in both survey and focus group data were the preparation required by tutors to facilitate sessions competently and the concept of being a ‘content expert’.

- *Time spent preparing for sessions*

Focus group discussions revealed that many tutors found the preparation time consuming and several tutors discussed the time spent revisiting the basic biomedical sciences. Others commented that while preparation for PBL is key, experience also plays a role in time spent becoming prepared.

*“I find you really need to be prepared before you go into each class. The more prepared you are the more confident you are on the topic.” (Focus Group 1)*

*“Personally I felt that if I didn’t know that stuff I would not be able to facilitate the group properly.” (Focus Group 1)*

b) *Content Expertise*

One of the single most asked questions by PBL tutors is ‘How deep do we need to go?’ This concern was expressed as a perceived lack of confidence by some tutors, while others were empowered by this lack of specialisation. Where some tutors felt less confident in their knowledge, this manifested in feelings of self-doubt and of being unprepared in focus group discussions.

*“How little, how much, that’s my biggest challenge.” (Focus Group 2)*

In contrast to the doubt expressed by some tutors, it is clear from our focus group discussions that, with experience, some tutors embrace the role of being a non-content expert facilitator and use this to reflect responsibility back to students with regard to their own self-directed learning and to following the PBL process.

*“It’s reassuring for them to know as well that we’re not expected to have all the answers.”*

*(Focus Group 2)*

##### 2. Engagement with PBL process

One of the most serious challenges that PBL tutors at GEMS face is students’ unauthorised access to the PBL cases and learning objectives (LOs) in advance of the tutorials, which can hinder the learning process. Tutors acknowledged this problem, with many suggesting potential solutions:

*“I would encourage them not to look at the LOs, even if they have them, until after we’re done because it defies the purpose of the whole process really. I think it worked ….if I knew which of the students had the LOs I’d find them least engaged.” (Focus Group 2)*

Another key feature of PBL is that groups should work together to activate and elaborate on prior knowledge. For the learning process to be successful, we believe it is important that tutors bring the focus back to the problem at hand. An effective tutor must also recognise when students are not actively engaging during PBL and use their facilitation skills to motivate students and bring their focus back to the group and to the learning outcomes of the session:

*“You have to be present and do it yourself.” (Focus Group 2)*

*“Just be aware if it’s going flat I think or if they’re particularly fatigued or not attentive...Try and switch things around and make it more interesting for them...” (Focus Group 2)*

Many of the tutors discussed the different milestones in the academic year that influence students’ engagement with the PBL process. These include the mid-semester changeover between groups and tutors and also the pre-exam periods which were noted as particular stressors.

*“I find once exam times begin to loom, the whole PBL process takes on quite a different dynamic” (Focus Group 2)*

*“You see a change probably about March …I think that’s probably the hardest time is to keep them engaged” (Focus Group 2)*

Tutors discussed their strategies for encouraging participation. One example refers to starting with a new PBL group:

*“I always have a ‘breaking the ice’ session, a discussion of advantages and disadvantages of PBL and learning styles at my first session with each group. These help to connect the students with each other and helps group dynamics and helps students to follow the PBL process…” (Focus Group 2)*

##### 3. Group dynamics

Issues addressed in the focus groups included the management of dominant / timid group members and the facilitation of a dysfunctional group.

The ability to manage these difficult personal interactions is complicated and requires skill and focus, even for the most experienced of tutors.

Techniques used to manage group dynamics included highlighting the value of good communication skills to enhance social congruence amongst the group.

*“I try and tell them that they are members of a team and as team members they have to practice both their participating and their listening skills” (Focus Group 1)*

Tutors expressed feelings ranging from self-doubt to empowerment and, with experience, they recognised that when given the time and space, groups were capable of managing and regulating themselves.

*“But the group is quite good at regulating itself often times actually...If you give it a little bit of space.” (Focus Group 2)*

- *Group challenges faced in PBL sessions*

As with any small group setting there are inherent issues and challenges that tutors are required to tackle.

i) *Dominant / Quiet Members of the Group*

Well-documented problems commonly experienced in PBL are the issues of students who are over contributing and those who are inherently quiet and or shy who need additional probing to speak up in class.

Tutors spoke about the challenges faced with both dominant and quiet group members, the impact of these personalities on group dynamics and the techniques used to limit their impact on the PBL process.

*“…You need firstly experience, but also you need to be able to deal with situations which arise in a diplomatic way that don’t insult the egos and the pride of the people who are adults themselves, you know.…Every time, no matter what it is, I just find it very challenging, I love it but I find it very challenging.” (Focus Group 2)*

ii) *Dysfunctional / Preformed Groups* 1

Several participants also spoke about managing groups (2 or more persons) who were disruptive and offered techniques used to tackle these issues:

*“Just give them the opportunity to mould the group themselves. Because I say to them it’s your PBL group so you want to get the most out of it and different things will work for different groups.” (Focus Group 2)*

Another aspect of group work tutors found challenging was that of entering a ‘pre-formed’ group. Tutors at GEMS join already formed groups mid-way through the semester in both Years 1 and 2. Many participants felt that students formed groups early in the semester and that these preformed groups could be difficult to engage with as part of the PBL session.

*“It’s difficult for the PBL process and you know because you’re this new person coming in on this, and they’re so used to doing it the way they did it.” (Focus Group 1)*

iii) *Access to Materials and Electronic Devices*

At the University of Limerick, PBL rules (See Additional file 1) prohibit access to secondary materials such as textbooks, notes and electronic devices during PBL session. Although this ban was introduced following discussion with the student body and tutors, it has been a source of contention for some students, while tutors favour the current rule.

*“…it's worked really well this year I think, that we have used no notes and no iPad. And we’re trusting our brains. When you go into a clinic, you can’t say excuse me I need to look it up, either you deal with it there or you say I don’t know, I’ll find out.” (Focus Group 2)*

*“You can’t have information at hand like that, it doesn’t work. And I find actually that once they have their computers, they become distracted”. (Focus Group 2)*

b) *Year-Specific Facilitation*

One theme which emerged from both survey and focus group data was the stark contrast of experiences felt by Year 1 and Year 2 tutors. The focus group discussions provided further insight into survey findings.

*“…I feel that second years are more confident and they can be more challenging for the tutor especially if you’re a replacement tutor. I think they probably have their own dynamic with their tutor and they know there are limits… I feel like I have to be more prepared and I have to be stricter somehow.” (Focus Group 2)*

*“For me the first year students are more eager I think to learn. And obviously it’s a new process for them.” (Focus Group 2)*

*“I love the confidence of year 2” (Focus Group 2)*

##### 4. Professional development

- *Role as facilitator*

The role of the PBL tutor demands a myriad of skills to enable students to take responsibility for their own small group learning while also providing guidance when required, especially to students who are new to PBL.

*“…. different personalities and trying to get them all to get on together and to achieve their learning outcomes together and no animosity in the group.” (Focus Group 2)*

*“…. you have to kind of give them some guidance but not control what they do, it’s kind of a balancing act I think.” (Focus Group 1)*

With experience, tutors described gaining a sense of confidence in their role and abilities to manage small group learning.

*“….It takes a while to find your feet and just kind of feel that you’re doing the right thing….We need to be reflective all the time on what we’re doing. But at least you also need to feel a certain amount of assuredness and confidence that what you’re doing is reasonably right. It mightn’t be exactly the same as everybody else, because everybody is different.” (Focus Group 1)*

b) *Benefits to clinical practice*

Tutors commented on the journey of being a PBL tutor moving from being a novice who perhaps had not studied the basic sciences for some time to learning significantly and where applicable, using new knowledge gained to help engage students in class but also add to their own clinical practice. Comments included:

*“Helps me to think outside the box when dealing with patients. Keeps me up to date”. (Survey)*

*“Has deepened my clinical/scientific knowledge of some conditions and broadened my differential diagnosis.” (Survey)*

*“Increased my knowledge, made me explore and reflect on communication skills, facilitation skills and how people learn”. (Survey)*

c) *Peer feedback and standardisation of practice*

Many of the tutors interviewed spoke about their need for standardisation of practice, peer audit and observation and the need to have opportunities to share their experiences with their peers. The perceived variety and need for conformity of practice was an idea highlighted by focus groups.

*“Everybody follows the format and yet we all have slightly different variations and interpretation of the exact same format. And you learn from your peer’s different problems that they’ve encountered, how they’ve dealt with them and I learn from that hugely…” (Focus Group 2)*

*“I think some effort should be made to find out if it’s standardised. I think we all don’t do it exactly the way that [author] would like us to do it, I suspect.” (Focus Group 1)*

Tutors expressed a willingness to participate in peer observation and for additional tutor meetings, which provide opportunities to discuss issues with cases or particular groups etc.

*“… I think we should have more meetings ……. if we can share our experiences it will help.” (Focus Group 2)*

*“If you kind of do it in a way and you say now I’m comfortable with the way I do it, well I better go and get uncomfortable and go and see how others do it.” (Focus Group 1)*

Feedback from students also aided in the professional development of tutors, allowing them to understand the needs and wants of students.

*“I always ask them what could I do differently? What would you like to see done differently in the group, how can we improve it, you know and get the feedback from them.” (Focus Group 1)*

##### 5. Rewards of tutoring

Participants overall had a very positive attitude towards PBL, noting their enjoyment of interacting with students and the opportunity to enhance learning without incurring clinical responsibility or having to ‘fix anyone’s problems’. Focus group comments also revealed positive attitudes and enthusiasm for tutoring.

*“…it is a privilege and it’s a pleasure actually to do it most of the time. And also it’s a challenge, so all that together is kind of, it’s very positive experience.” (Focus Group 1)*

*“I love bringing back what they’re learning to the case and then that they see, oh that’s why his blood pressure is up.” (Focus Group 2)*

## Discussion

This study examined the experiences of PBL tutors in a small group learning environment at the Graduate Entry Medical School at the University of Limerick. Our findings reveal that tutors in our PBL programme share many similar experiences to those of previously reported studies with common themes including the challenges of managing group dynamics, the development of confidence in tutoring with experience and a willingness to learn from peers to improve practice. The results of this study also identify several less commonly discussed issues including the impact of the use of technology on student engagement during PBL and the positive effects of being a PBL tutor for practising clinicians.

The issue of managing group dynamics is one which has been widely researched in the area of PBL and small group learning [29] and our tutors report similar experiences to those previously described. The differences between teaching and facilitating and the difficulty of judging ‘when and how’ to intervene, as previously described by Spronken-Smith and Harland [15] and others [9, 14, 30] were also raised in the present study. Tutors spoke about the need to balance the amount of input required and the struggle between guiding students to keep them on track while avoiding acting as a teacher. Student behaviours that impede the PBL process such as over-contributing or dominant students as well as reticent students who do not fully contribute were all discussed. It is clear from our tutors that learning from their previous experiences and using their communication skills enabled them to deal with challenging groups or students.

In dealing with issues such as group dynamics that influence the success of PBL, the role of a PBL tutor is very different to that of a traditional teacher or tutor and requires a unique set of skills [31]. A common discussion with regard to the recruitment and training of PBL tutors is the level of content expertise required to be an effective tutor, with ‘expertise’ being categorised as either having previous experience of PBL or, alternatively, being a subject matter expert [21, 32]. All of the tutors in our study are qualified medical practitioners and would therefore be classed as ‘content experts’ according to several definitions [11, 22, 33]. However participants described their ongoing difficulties in knowing how much content expertise is sufficient with regard to the amount of preparation needed for sessions and their own confidence in facilitating sessions. Even the more-experienced tutors spent a comparable amount of time preparing for tutorials to the less-experienced tutors (Fig. 1). Tutors reported that experience gained with practice led to a feeling of greater preparedness and consequently, enhanced their confidence in their abilities as a tutor. This finding resonates with previous studies which acknowledged the importance of experience in the development of PBL tutors’ attitudes and skills [9, 14, 15].

The uncertainties and ambiguities experienced by tutors with regard to their own content expertise, the depth and breadth of information required and their performance as facilitators suggests an interesting comparison with reported student experiences of PBL [34, 35]. The ambiguity of what is required and expected of students [34] is reflective of tutor’s own experiences. Students new to PBL often discuss their misunderstanding of the tutor’s role and participants in the present study have highlighted this issue - their need to provide direction to students but also to act as group facilitator [22]. Many of these parallels can be drawn quite easily but to the best of the authors’ knowledge, no documented studies have been completed which do so. Awareness of this observation may lead to tutors having a greater shared social congruence with their students, a characteristic that contributes to tutor effectiveness [8, 22, 36].

Confidence as a tutor was also highlighted by comments on the disparity between what is seen as the ‘gold standard’ of PBL practice and what is actually practiced. Many tutors observed that they ‘should be’ doing things a certain way, in accordance with their tutor training, but that what is actually practised may be different. This finding echoes the observation by Spronken-Smith and Harland [15] that tutors felt bound to adhere to a strict set of PBL rules to ensure standardisation of learning outcomes for their students. One way to encourage standardisation of practice is through peer observation and audit, as suggested by some of the tutors in this study. While novice tutors are audited by experienced faculty and all tutors receive regular feedback from students, the willingness of tutors for increased peer observation is similar to previous studies [16, 30] which have noted the importance of peer support and the fostering of a ‘community of practice’ in PBL tutor development. This finding can be acted upon by providing more opportunities for interaction and observation between tutors along with the regular scheduled meetings to discuss cases and share experiences. In addition to the request for peer feedback, it is also encouraging that our tutors reported reflecting on their own practice frequently (Table 2) which contrasts with the study by Maudsley [14] which reported that only 2 tutors out of 34 identified the importance of the reflective nature and skills required by PBL tutors.

One of the novel findings identified by this study was the issue of students’ prior access to PBL cases and learning objectives and its influence on student engagement. This was very apparent in undermining the entire PBL process and, apart from anecdotal reports of this issue, we are not aware of it having been extensively described in the literature. Zhang et al. [37] addresses medical students’ use of the ‘informal curriculum’ – sharing of previous years peer notes as a method of learning. The majority of students (86%) had access to notes and perceived this to be of benefit to their own informal learning in terms of saving time in learning, ensuring they cover all the topics required and focusing on what is important to pass exams and become a good doctor [38]. This perceived value of having access to notes is apparent but authors would argue that this is the opposite to the values of self-directed learning and PBL. Another issue affecting PBL engagement identified in this study was the effect of the time of year with end-of-semester periods preceding exams proving problematic for tutors as students’ attention and attendance to class waned. This issue is one that tutors and faculty can recognise as an important factor influencing PBL group dynamics and participation and is also a factor that may introduce variance in research studies on PBL. These findings highlight issues that need further investigation to develop strategies to overcome them and improve engagement with PBL.

Another factor affecting student engagement identified in this study is that of the differences between students of Years 1 and 2 of the medical degree course. During the period covered by the survey, Year 1 tutors reported considerably more positive experiences with their groups, greater discussion of cases and were more satisfied with group performance compared to Year 2 tutors (Fig. 2). However, it is clear from focus group discussions that tutors of both years had experienced difficulties. This difference between survey and focus group data may reflect the fact that every PBL group is different and reminds us that research findings on PBL student-tutor interactions may vary substantially depending on the cohorts studied. For this reason, it is important for research on PBL to ensure that comparisons are drawn between groups in the same academic year.

With increasing use of technology in education, an emerging theme is the introduction of mobile devices to PBL. In our programme, while students are obliged to own iPads to access learning resources for certain aspects of the course, their use during PBL has resulted in students being less engaged. The use of electronic devices during PBL tutorials is therefore prohibited in an effort to promote full engagement with the collaborative PBL process. This has affected group dynamics during PBL in two ways: firstly, students who felt they should be allowed to use devices during PBL were less engaged and their distraction disrupted group dynamics and secondly, the conflict between tutors and students who challenge the ban is also disruptive. In this study, it is clear that our tutors are still in favour of retaining the ban on use of devices and notes during PBL as the positive effect this has on students’ engagement during PBL outweighs the negative effect of the conflict it causes with a minority of students. These findings contrast with Wood et al. [39] who report that use of mobile devices during PBL in a hybrid-PBL medical curriculum was perceived by tutors to improve information gathering by students but also to have altered the PBL process. Sundvik et al. [40] have also reported largely positive attitudes of tutors to the introduction of electronic devices during PBL for note-taking and information retrieval and they report a positive effect on group dynamics. Chan et al. [22] have developed recommendations for the use of mobile devices during PBL which address facilitators’ concerns about the potential for distraction and that the use of devices favours superficial learning. In our opinion, activation of prior knowledge is central to PBL and reliance on electronic devices during PBL, as for notes or textbooks, is likely to detract from this process.

The importance of professional development has been highlighted positively by tutors in this study - upskilling in terms of their own knowledge helps improve tutor’s own skills base and promotes openness to the changing learning environment. An interesting finding in our study was the tutors’ perception of how facilitating PBL impacted their clinical practice. Tutors reported that PBL facilitation experience improved reflection, updated basic biomedical science knowledge and enhanced clinical reasoning, all of which benefitted their clinical practice. Lockyer et al. [41] reported that more involvement in clinical teaching was associated with higher clinical performance ratings but from our review of the literature little if anything has been reported on the impact of facilitating PBL in the basic biomedical sciences on clinical practice. Furthermore, the overall positive attitudes and enjoyment of PBL demonstrate that tutoring Year 1 and 2 students is a rewarding experience for practising clinicians.

Whilst some of these findings can be seen as local and useful (such as the impact of exams on students’ engagement, ban on use of electronic devices and access to materials at the Graduate Entry Medical School), there are some which can have a far reaching effect on PBL at other medical schools. The issue of access to learning objectives (LOs) is a novel finding which to the best of the authors’ knowledge has yet to be documented in literature. There are many different approaches which can be taken to limit or stop the sharing of LOs from peers, some schools have adopted a stance of making weekly LOs available on demand prior to class, a practice which authors would argue is not in line the true teachings of PBL. This issue is one which authors would argue needs to be addressed at a local level but also internationally.

The improvement of clinician’s skills related to their facilitation of PBL sessions is a factor which also has some far reaching consequences both locally and internationally. Whilst there is no literature to support this, authors would argue that if tutors (whether medically trained or specialised) are agreeing that facilitation of PBL group sessions is improving their clinical practice, there should be a call for more potential tutors to become involved and become a tutor. Where tutors are required to recall and refresh their medical knowledge, this ensures that students are adequately supported but also a clinician’s patients are feeling the positive effects of this facilitation. This additional benefit does add value for tutor’s own professional and personal development.

### Study strengths and limitations

Strengths of this study include the high response rate (89%) for survey participation. A limitation of this study was that the survey data related to a finite period of two modules while focus group data reflects the overall tutor experience. Our findings identify the time of academic year as an important factor influencing student engagement in PBL and consequently, tutors’ experiences of PBL. This should be taken into account when interpreting and comparing the results of studies completed at different times of the year. We suggest that future work should capture opinions longitudinally to ensure a comprehensive view.

## Conclusions

Tutors provide valuable insights into factors affecting student engagement which will aid in promoting student motivation and successful outcomes of PBL. Our findings suggest that experience gained with time and with facilitating a diverse range of PBL groups considerably enhances the confidence and skills of PBL tutors and that sharing experience is a valuable part of professional development as a PBL tutor. The results of this study will inform PBL practices and procedures with regard to tutor recruitment, training and support to enhance the experience of PBL for tutors and students.

## Additional files

Additional file 1: Context: PBL at GEMS. Description of data: Further context to PBL at the Graduate Entry Medical School. (DOCX 16 kb)

Additional file 2: PBL tutor survey. Description of data: Full length survey that was utilised within the study by PBL tutors. (DOCX 32 kb)

Additional file 3: Focus Group Guide. Description of data: Focus Group Interview Guide that was utilised as part of this study. (DOCX 14 kb)

## Abbreviations

GEMS: Graduate Entry Medical School
LOs: Learning objectives
PBL: Problem-based learning
UL: University of Limerick

## Footnotes

1 Dysfunctional/Preformed Groups: PBL groups (at GEMS) change tutors at specific time points in the semester. Tutors therefore face groups that have already been together or preformed for some time which can results in issues.
