# Supplementary material for: What can we learn from problem-based learning tutors at a graduate entry medical school? A mixed method approach
Source: BMC Med Educ. 2018 May 4;18:96. doi: 10.1186/s12909-018-1214-2 (PMC5935969; doi:10.1186/s12909-018-1214-2)
Supplement: Supplementary file 1 — Context: PBL at GEMS. Description of data: Further context to PBL at the Graduate Entry Medical School. (DOCX 16 kb) [file 12909_2018_1214_MOESM1_ESM.docx]

#### Additional file 1- Context: PBL at GEMS

1. *The Ground Rules*

At GEMS there are ten ground rules for PBL groups, a contract in effect. Tutors and students are required to discuss and sign up for the rules when the group is formed. This contract can then be used to highlight the rules should a student breach the contract, e.g a student over contributing and decide on an approach to be taken. Groups may have additional rules and review these rules periodically to see how well these rules are being adhered to.

**The 10 Ground Rules of Problem-based Learning in UL**

1. Begin at the agreed time
2. Come prepared
3. Be professional
4. Be committed to the success of the group process
5. Conduct only one conversation at a time
6. Listen
7. Everyone’s opinion are important
8. No electronic devices during PBL
9. No texts (except dictionary and BNF) or reading from notes (case summary exception) allowed during tutorials
10. Support one another in the struggle and enjoy!

*2. Roles and Responsibilities*

The role of PBL tutors is to facilitate group sessions and create a supportive environment where students challenge and question each other constructively. Some of the tutors’ responsibilities include:

- Facilitation of discussion
- Asking open ended questions when needed to encourage group discussion
- Enhancement of use of educational tools during tutorials
- Providing suggestions
- Summarising key points raised in the discussion
- Helping in the creation of an open, healthy environment that encourages group discussion, experimentation and dealing with uncertainty
- Providing feedback to the groups at the end of the case and receive feedback from the group
- Monitoring students’ progress
- Running group assessment

PBL tutors at UL also act as academic advisors to students in their PBL group. When a group changes a tutor they also change academic advisor. The tutor provides support and guidance should the student experience academic difficulties.

*3. Tutor Training & Meetings*

Annual training days are provided for all PBL tutors with new tutors required to attend. Following the end of each module a tutor meeting takes place, allowing tutors to interact and discuss any issues, with cases or groups, with colleagues. Attendance of a minimum 50% of tutor meetings across an academic year is required.

*4. Tutor Backgrounds*

All the PBL tutors at the GEMS in UL are fully qualified medical doctors and many still practicing clinicians. Tutors work in various specialised areas and whilst may not be ‘content experts’, many hold an extensive experience acting as a PBL tutor; tutors at the GEMS hold between a minimum of one year experience with some having up to nine years’ experience acting as a PBL tutor.
