# Supplementary material for: What can we learn from problem-based learning tutors at a graduate entry medical school? A mixed method approach
Source: BMC Med Educ. 2018 May 4;18:96. doi: 10.1186/s12909-018-1214-2 (PMC5935969; doi:10.1186/s12909-018-1214-2)
Supplement: Supplementary file 2 — PBL tutor survey. Description of data: Full length survey that was utilised within the study by PBL tutors. (DOCX 32 kb) [file 12909_2018_1214_MOESM2_ESM.docx]

#### Additional file 2- PBL Tutor Survey


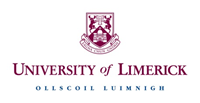


**PBL Tutor Survey– to be completed at the end of each module**

**DATE:**

**MODULE:**

1) Life Cycle

2) Life Support 2

3) Life Protection 1

4) Life Control 2

**Year group:**

*Tutor Background*

1. How long have you been a GEMS PBL tutor for?

a. <12 months

b. 12-24 months

c. >2 years

2. Which PBL sessions do you facilitate?

a. Year 1

b. Year 2

c. Both Year 1 and Year 2

3. What is your clinical background (*i.e.* speciality)?

4. Are you still working in clinical practice?

5. If you answered yes to the above (Q6.) please let us know how many sessions on average you work in clinical practice per week?

a. <5 sessions

b. 5-10 sessions

c. >10 sessions

6. When was the last time you undertook a GEMS PBL tutor training course?

7. Have you completed any formal post-graduate training in education?

8. If you answered yes to the above (Q9.) please let us know what type of training you have completed?

9. Are you registered on any formal post-graduate training in education course?

10. If you answered yes to the above (Q11.) please let us know what type of course you are registered on (*i.e.* award, certificate, diploma or degree level)

For the following statements please be sure to read each statement carefully and then select the most appropriate response in each case

11. How many PBL sessions did you facilitate during this module?

a. <5 sessions

b. 5-10 sessions

c. >10 sessions

12. Have you facilitated this module before?

a. Yes

b. No

13. On average how much time did you spend preparing for this module?

<30 mins <60 mins 60-90 mins 90-120 mins >120 mins

14. How effective was this module in stimulating student learning?

Extremely Effective Quite Effective Slightly Effective Moderately Effective Not At All

15. How positive has your experience been for this module?

Very Positive Positive Neither positive or negative Negative Very Negative

16. How useful were the tutor guides for this module?

Very useful Quite useful Moderately useful Somewhat useful Not at all useful

17. How useful were the resources for this module?

Very useful Quite useful Moderately useful Somewhat useful Not at all useful

18. How confident were you in using the information technology (IT) needed during a PBL session in this module?

Extremely confident Quite confident Moderately confident Slightly confident Not at all confident

19. In your opinion how relevant is this module for students at this point in the curriculum?

Very relevant Quite relevant Moderately relevant Slightly relevant Not at all relevant

20. In your opinion how relevant is this module for newly qualified doctors?

Very relevant Quite relevant Moderately relevant Slightly relevant Not at all relevant

21. Are the cases in this module on the whole well-designed?

Very well-designed Well-designed Neither well-designed or poorly-designed Poorly-designed Very poorly-designed

22. Are the cases in this module on the whole well-sequenced?

Very well-sequenced Well-sequenced Neither well-sequenced or poorly-sequenced Poorly-sequenced Very-poorly sequenced

23. In your opinion how reflective are the cases in this module of up-to-date clinical practice?

Very reflective Quite reflective Moderately reflective Slightly reflective Not at all reflective

24. In your opinion how easily did cases in this module activate the students’ prior knowledge?

Very Easily Quite Easily Moderately Easy Slightly Easy Not at all Easy

25. How much discussion did the cases provoke in this module?

A great deal Quite a bit Some A little bit Almost none

26. How much input from you was required for the students to generate the relevant Learning Objectives?

A great deal Quite a bit Some A little bit Almost None

27. How often in this module did you discuss the ground rules?

Every session Every Week Fortnightly Once per module Never

28. In order to improve my performance I reflect on my role as a facilitator:

Every session Every week Fortnightly Once per module Never

29. Has your experience as a PBL tutor impacted you as a clinician? If so, how:

|  | 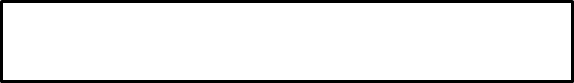 |
| --- | --- |
|  |  |

30. How often did you encourage your group to reflect on group dynamics and effective working?

Every session Every week Fortnightly Once per module Never

31. How much input from you was required to maintain good group dynamics?

A great deal Quite a bit Some input A little bit Almost none

32. How satisfied were you that the group worked well together?

Extremely satisfied Quite satisfied Moderately satisfied Slightly satisfied Not at all satisfied

33. How often did individuals within the group come to you with concerns about the group?

Very often Often Sometime Once in a while Never

34. Were there any particular difficulty(ies) that arose in the course of the sessions? If yes please outline below.

35. If applicable how did you overcome these difficulties?

|  | 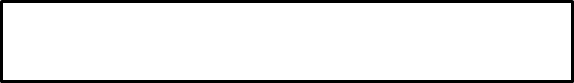 |
| --- | --- |
|  |  |

36. Please comment on anything that you think worked well for you or the students in this module.

|  | 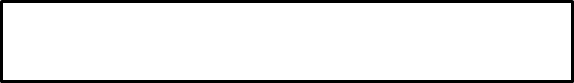 |
| --- | --- |
|  |  |

Following this questionnaire we are hoping to complete two focus group interviews. These focus groups will be made up of tutors only and will give you the opportunity to discuss topics highlighted today in more detail. If you would like more information or would like to participate in a future focus group please leave your email address or relevant contact details below.

*Additional comments*

Please let us know how you think PBL is working at GEMS:

| \|  \| \| --- \| |
| --- | --- |

Please let us know how you think PBL could be improved at GEMS

|  |  |
| --- | --- |
|  | \|  \| \| --- \| |

Thank you for completing our questionnaire!

This research study has received Ethics approval from the Education and Health Sciences Research Ethics Committee (quote approval number). If you have any concerns about this study and wish to contact someone independent you may contact: EHSREC Approval Number: 2016_06-23_EHS

Chairman Education and Health Sciences Research Ethics Committee

EHS Faculty Office

University of Limerick

Tel (061) 234101
