# Supplementary material for: What can we learn from problem-based learning tutors at a graduate entry medical school? A mixed method approach
Source: BMC Med Educ. 2018 May 4;18:96. doi: 10.1186/s12909-018-1214-2 (PMC5935969; doi:10.1186/s12909-018-1214-2)
Supplement: Supplementary file 3 — Focus Group Guide. Description of data: Focus Group Interview Guide that was utilised as part of this study. (DOCX 14 kb) [file 12909_2018_1214_MOESM3_ESM.docx]

#### Additional file 3: Focus Group Interview Guide

| **Number** | **Question** |
| --- | --- |
| **1** | How did you come to be a PBL tutor here in GEMS? |
| **2** | How would you describe your role as a PBL tutor? |
| **3** | How well do you feel prepared in your role as tutor? |
| **4** | What are some of the challenges you face as a facilitator? |
| **5** | Are there any approaches that you adopt to face different challenges? |
| **6** | Is there anything you know now as a tutor that you didn’t starting off as a new tutor? |
| **7** | How you do keep students engaged in the PBL process? |
| **8** | What aspect of being a PBL tutor do you find most rewarding? |
| **9** | In what way do you encourage successful teamwork within the group? |
| **10** | In what way could PBL at GEMS in UL be further improved in your opinion? |
